# Assessing blood oxygen level–dependent signal variability as a biomarker of brain injury in sport-related concussion

**DOI:** 10.1093/braincomms/fcad215

**Published:** 2023-08-16

**Authors:** Evan D Anderson, Tanveer Talukdar, Grace Goodwin, Valentina Di Pietro, Kamal M Yakoub, Christopher E Zwilling, David Davies, Antonio Belli, Aron K Barbey

**Affiliations:** Decision Neuroscience Laboratory, University of Illinois Urbana-Champaign, Urbana, IL 61801, USA; Air Force Research Laboratory, Wright-Patterson AFB, OH 45433, USA; Decision Neuroscience Laboratory, University of Illinois Urbana-Champaign, Urbana, IL 61801, USA; Decision Neuroscience Laboratory, University of Illinois Urbana-Champaign, Urbana, IL 61801, USA; Department of Psychology, University of Nevada, Las Vegas, NV 89557, USA; Decision Neuroscience Laboratory, University of Illinois Urbana-Champaign, Urbana, IL 61801, USA; Neurotrauma and Ophthalmology Research Group, Institute of Inflammation and Ageing, College of Medical and Dental Sciences, University of Birmingham, Birmingham B15 2TT, UK; NIHR Surgical Reconstruction and Microbiology Research Centre, University Hospitals Birmingham NHS Foundation Trust, Birmingham B15 2TH, UK; Neurotrauma and Ophthalmology Research Group, Institute of Inflammation and Ageing, College of Medical and Dental Sciences, University of Birmingham, Birmingham B15 2TT, UK; NIHR Surgical Reconstruction and Microbiology Research Centre, University Hospitals Birmingham NHS Foundation Trust, Birmingham B15 2TH, UK; Decision Neuroscience Laboratory, University of Illinois Urbana-Champaign, Urbana, IL 61801, USA; Neurotrauma and Ophthalmology Research Group, Institute of Inflammation and Ageing, College of Medical and Dental Sciences, University of Birmingham, Birmingham B15 2TT, UK; NIHR Surgical Reconstruction and Microbiology Research Centre, University Hospitals Birmingham NHS Foundation Trust, Birmingham B15 2TH, UK; Neurotrauma and Ophthalmology Research Group, Institute of Inflammation and Ageing, College of Medical and Dental Sciences, University of Birmingham, Birmingham B15 2TT, UK; NIHR Surgical Reconstruction and Microbiology Research Centre, University Hospitals Birmingham NHS Foundation Trust, Birmingham B15 2TH, UK; Decision Neuroscience Laboratory, University of Illinois Urbana-Champaign, Urbana, IL 61801, USA; Department of Psychology, University of Illinois, Urbana, IL 61801, USA; Department of Bioengineering, University of Illinois, Urbana, IL 61801, USA; Center for Brain, Biology, and Behavior, University of Nebraska-Lincoln, Lincoln, NE 68588, USA

**Keywords:** mild traumatic brain injury, concussion, sport-related concussion, neuroimaging, BOLD variability

## Abstract

Mild traumatic brain injury is a complex neurological disorder of significant concern among athletes who play contact sports. Athletes who sustain sport-related concussion typically undergo physical examination and neurocognitive evaluation to determine injury severity and return-to-play status. However, traumatic disruption to neurometabolic processes can occur with minimal detectable anatomic pathology or neurocognitive alteration, increasing the risk that athletes may be cleared for return-to-play during a vulnerable period and receive a repetitive injury. This underscores the need for sensitive functional neuroimaging methods to detect altered cerebral physiology in concussed athletes. The present study compared the efficacy of Immediate Post-concussion Assessment and Cognitive Testing composite scores and whole-brain measures of blood oxygen level–dependent signal variability for classifying concussion status and predicting concussion symptomatology in healthy, concussed and repetitively concussed athletes, assessing blood oxygen level–dependent signal variability as a potential diagnostic tool for characterizing functional alterations to cerebral physiology and assisting in the detection of sport-related concussion. We observed significant differences in regional blood oxygen level–dependent signal variability measures for concussed athletes but did not observe significant differences in Immediate Post-concussion Assessment and Cognitive Testing scores of concussed athletes. We further demonstrate that incorporating measures of functional brain alteration alongside Immediate Post-concussion Assessment and Cognitive Testing scores enhances the sensitivity and specificity of supervised random forest machine learning methods when classifying and predicting concussion status and post-concussion symptoms, suggesting that alterations to cerebrovascular status characterize unique variance that may aid in the detection of sport-related concussion and repetitive mild traumatic brain injury. These results indicate that altered blood oxygen level–dependent variability holds promise as a novel neurobiological marker for detecting alterations in cerebral perfusion and neuronal functioning in sport-related concussion, motivating future research to establish and validate clinical assessment protocols that can incorporate advanced neuroimaging methods to characterize altered cerebral physiology following mild traumatic brain injury.

## Teaser

Anderson *et al*. report that blood oxygen level–dependent signal assessed by functional MRI outperforms a standard post-concussion neurocognitive test for classifying concussed athletes and predicting post-concussion symptoms. Blood oxygen level–dependent signal variability may hold promise as a neurobiological marker for alterations in cerebral perfusion and functioning following concussion.

## Graphical Abstract

**Graphical Abstract:**
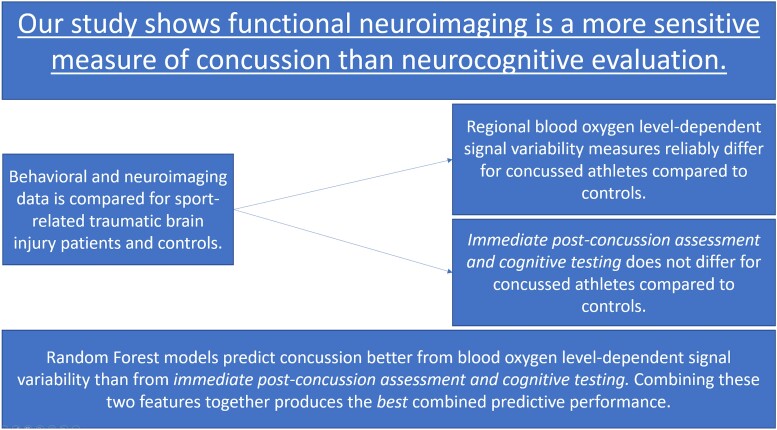

## Introduction

Traumatic brain injury (TBI) is a prevalent neurological disorder in the USA, leading to significant rates of short- and long-term sequelae among young athletes^1^ and military service members.^2^ Mild TBI (mTBI)—accounting for 80–90% of all brain injuries—is emotionally, physically and financially taxing to patients and families and represents a substantial economic burden to the healthcare system.^3^ mTBI is a traumatically induced physiological disruption of neurological function, diagnosed as loss of consciousness (LOC) of 30 min or less, memory loss or amnesia (retrograde or anterograde) for 24 h or less, any alteration in mental state, any focal neurologic deficits and/or an initial Glasgow Coma Scale (GCS) score of 13–15.^3^ mTBI induces persistent symptoms in 10–20% of individuals, with a minority experiencing persisting symptoms.^2,4^ Sport-related concussion (SRC) is a prevalent subtype of mTBI that involves a blow to the head (directly or indirectly) during athletic play that results in a rapid onset of neuropathological change that leads to a diverse range of symptoms.^5^ Given the variability in injury mechanisms, SRCs are among the most complex injuries in sports medicine to diagnose.^5^

Clinicians rely on a variety of assessments to diagnose SRC, including brief neurological examinations, symptom checklists, neurocognitive tests (e.g. Immediate Post-concussion Assessment and Cognitive Testing; ImPACT)^6,7^ and, occasionally, clinical neuroimaging when severe structural abnormalities are suspected.^3^ While informative, these assessments are limited in their ability to assess the presence and extent of injury or identify neurophysiologically vulnerable individuals. Athletes with minimal physiological and neurocognitive symptoms may therefore be cleared to return-to-play prematurely, which can exacerbate symptoms, increase risk for severe secondary injuries and complicate or prolong recovery.^2,4,8^ Thus, current research aims to discover neurobiological markers that can be used to objectively assess the presence and severity of SRC and to determine when the brain has recovered sufficiently to permit safe return-to-play.

### Pathophysiology and neuroimaging of mTBI

Concussion symptoms reflect a complex series of neurometabolic events that occur during and after the initial impact. Physiological damage from SRC typically occurs in stages, starting with the initial impact or change in velocity (i.e. acceleration/deceleration and inertial rotation caused by blow to the head or indirectly to the body) and subsequent structural deformation.^9^ Acute biomechanical injury—sudden stretching of neuronal and axonal membranes—triggers a cascade of neurometabolic changes including the abrupt release of neurotransmitters, ionic fluxes and unregulated extracellular glutamate release, ultimately leading to hyperglycolysis and axonal injury or cell damage.^8^ This intricate ‘neurometabolic cascade’^8^ can manifest sequelae including dizziness, nausea, headache, insomnia, amnesia, attentional issues and other physiological and neurocognitive symptoms.^10,11^

Several brain regions, including anterior frontal and temporal areas, medial temporal structures, corpus callosum and subcortical white matter pathways, are particularly vulnerable to bruising and injuries caused by inertial forces.^3,9,12,13^ Repetitive TBI in these areas is a particularly serious concern in SRC, as secondary blows that occur during this vulnerable metabolic window can produce catastrophic secondary impairments,^14^ highlighting the need for accurate diagnostics for safely clearing individuals to return to normal activity following a concussion.

As neurobiological correlates of cognitive and mental health dysfunction from SRC are difficult to detect with standard axial neuroimaging techniques (i.e. CT and structural MRI),^15^ researchers have increasingly utilized more advanced functional neuroimaging techniques to elucidate underlying changes in neuropathology post-injury.^12,16^ Functional MRI (fMRI) has shown promise for characterizing alterations in neural function post-mTBI across multiple domains. Task-based fMRI studies have demonstrated changes in activation in the parietal cortex, dorsolateral prefrontal cortex and the hippocampus in concussed athletes relative to healthy controls.^17^ Further, functional connectivity assessed from resting-state fMRI revealed altered connectivity within the default mode, fronto-parietal and motor-striatal networks relative to controls in individuals with mTBI.^18,19^ fMRI methods may provide objective biomarkers of neurophysiological injury, affording greater sensitivity and specificity for SRC diagnoses. Recent research suggests that while structural imaging and neurocognitive tests did not accurately discriminate mTBI patients from controls, resting-state functional connectivity measures correctly classified SRC status and predicted long-term cognitive sequealae.^20^ Functional neuroimaging methods may facilitate SRC evaluation through identification of altered haemodynamics and neurometabolism, detecting impaired neurovascular coupling, cerebral blood flow (CBF) and oxygen metabolism associated with abnormal brain function.^21^

### Blood oxygen level–dependent variability

Neuroscience evidence demonstrates that regional brain activity is inherently variable over time^22,23^ and that patterns of variability as measured from blood oxygen level–dependent (BOLD) signal are closely linked to neural information processing, cognitive function and brain health.^24,25^ Recent studies have demonstrated that BOLD variability can detect vascular pathologies in cerebral small vessel disease,^26^ Alzheimer’s disease,^27^ post-traumatic stress disorder^28^ and stroke^29^ by capturing underlying differences in cerebrovascular compliance^26^ that alter haemodynamic function and neurovascular coupling, motivating the investigation of BOLD variability for detecting neurophysiological alterations in mTBI.

Mechanisms that may alter BOLD variability following mTBI may include changes in cerebral perfusion,^30,31^ increased reactivity of smooth muscle in microvessel walls^32^ and reduction in the density and diameters of capillaries both proximal and distal to the site of injury.^33^ Furthermore, altered BOLD variability can indicate sites of neural proliferation and synaptogenesis, indexing post-mTBI neuroplasticity that drives changes in neural signalling and BOLD activation during recovery.^15^ BOLD variability may therefore serve as a biomarker of changes in individuals’ cerebral vascular status during and following traumatic neurophysiological insult, potentially assisting in the diagnosis of SRC.

### The present study

The aim of the present study is to compare regional measures of BOLD variability and cognitive assessment scores for their complementary roles in the evaluation of SRC. Neuroimaging indices of BOLD variability may represent a novel lens for objectively evaluating SRC status by detecting functional disruption, providing information beyond conventional neuropsychological assessment batteries that may improve the detection and management of concussion pathology. While clinically informative, neurocognitive assessments remain limited in their ability to assess the extent of injury or identify neurophysiologically vulnerable individuals. To investigate the potential role of neurocognitive testing and functional neuroimaging in SRC diagnosis, we examined group differences in regional BOLD signal variability and neurocognitive performance on ImPACT in athletes diagnosed with SRC (compared against age-matched control athletes without SRC). We then applied a supervised machine learning algorithm (bagged ensemble random forests) to assess whether whole-brain BOLD variability measures, ImPACT module scores or BOLD and ImPACT composite scores together can discriminate healthy, concussed and repetitive concussion patients. We further applied bagged random forests to assess the extent to which regional BOLD variability measures and ImPACT scores in isolation or combination accurately predict clinical symptom load. Our study therefore assessed BOLD variability in both classification and regression machine learning frameworks for detecting SRC status or post-concussion symptoms from functional neuroimaging data (either in comparison or combination with neuropsychological assessment).

## Materials and methods

### Study approval

This study was carried out in accordance with the recommendations and approval of the University of Birmingham Research Ethics Committee. The protocol was also approved by the National Institute of Health Research Centre for Surgical, Reconstruction and Microbiological Research Centre (NIHR SRMRC—Ethics Ref. 11-0429AP28). All subjects gave written informed consent in accordance with the Declaration of Helsinki. Deidentified data from this cohort may be made available by the authors on request.

### Participants

Study participants were recruited through the Surgical Reconstruction and Microbiology Research Centre (SRMRC), based at Queen Elizabeth Hospital of Birmingham (United Kingdom), as part of the repetitive concussion in sport (RECOS) study.^34^ Participants included 29 semi-professional rugby athletes diagnosed with SRC and 6 additional rugby athletes diagnosed with repetitive SRC over a 21-day window. Athletes were excluded if they required hospital admission after initial assessment for concussion; presented with intracranial blood, brain tissue injury or non-concussion–related pathologies on initial CT/MR scan; or had history of neurodegenerative pathology or chronic alcohol or drug abuse. In addition, 15 age-matched controls who have not received a concussion in the previous 3 months were enrolled. Other study design features are reported in Yakoub *et al*.^34^

### Neuroimaging data acquisition and analysis

All data were collected on a 3 Tesla Philips Achieva MRI scanner in Birmingham University Imaging Centre (BUIC). A high-resolution multi-echo T_1_-weighted magnetization-prepared gradient echo structural image was acquired for each participant [0.9 mm isotropic, repetition time (TR) = 800 ms, echo time (TE) = 30 ms, with sensitive encoding parallel acquisition (SENSE)].

The functional neuroimaging data were acquired using an accelerated single-shot gradient echo echoplanar imaging (EPI) sequence^35^ sensitive to BOLD contrast [3.0 × 3.0 × 3.0 mm voxel size, ascending acquisition with no slice gap, TR = 2 ms, TE = 35 ms, field of view (FOV) = 240 mm, 80° flip angle, total 6 min acquisition].

During the resting-state fMRI scan, participants were asked to keep their eyes closed. Visual contact could be established at any time to the control room via a coil-mounted reverse mirror. All participants were instructed to lie still, keep their eyes closed and to try and think of nothing.

### MRI pre-processing analysis

All MRI data processing was performed using containerized processing pipelines for reproducible analysis of neuroimaging data. Pre-processing steps were performed using *fMRIPrep* 20.2.0^36,37^ and XCP Engine 1.0.^38,39^ Pre-processing entailed slice timing correction, motion correction, spatial smoothing [3 mm full width at half maximum (FWHM) kernel], nuisance signal regression, temporal bandpass filtering, linear registration of functional images to structural images and non-linear registration of structural images to a standard-space MNI152 brain template (2 mm isotropic voxel resolution).

Head motion parameters were accounted for using independent component analysis with automatic removal of motion artefacts (ICA-AROMA) analysis.^40^ All nuisance variables were modelled via a single generalized linear model (GLM), to remove spurious correlations and noise introduced by head motion and variables of no interest. In addition to ICA-AROMA components classified as noise, these included head motion correction parameters, individual volume motion outliers estimated using DVARS^41^ with outliers flagged above 1.5 standardized DVAR, framewise displacement exceeding 0.5 mm and mean white matter and cerebrospinal fluid signals averaged across all voxels identified from the segmentation of the high-resolution magnetization-prepared rapid gradient echo (MPRAGE). The fully pre-processed resting-state fMRI data were taken as the residuals from this GLM model. The residual image was transformed into normalized MNI152 space and resampled to 4 mm isotropic voxels.

### BOLD variability

Resting-state mean square successive difference (MSSD)^42,43^ BOLD signal variability was computed as the standard deviation of successive differences in the time-series signal extracted for each of the 200 grey matter regions defined by the Schaefer parcellation atlas.^44^ MSSD mean-centres the amplitude of difference in BOLD signal between frames, providing a more reliable metric of BOLD variability than standard deviation by avoiding inflated estimates of variability. The Schaefer 200 atlas provided sufficient spatial resolution and functional homogeneity within each parcel for examining regional MSSD_BOLD_ signal across the entire cortex.

### Computerized neurocognitive assessment

Participants completed the 25 min computerized ImPACT assessment under identical administration conditions. ImPACT is a multi-domain online neuropsychological test that has been used as a standard technique documenting baseline cognitive function, characterizing the effects of concussive injury and monitoring the progress of recovery.^45^ The test was designed in the early 1990s specifically to assess concussed players of the National Football League^46^ and is now a Food and Drug Administration (FDA)–approved neuropsychological testing tool in SRC.^47^ Inclusion of this particular cognitive test has been demonstrated to increase the sensitivity of post-concussion assessment beyond symptomatic evaluation and physical examination.^48^

ImPACT includes a demographic survey, a brief medical history questionnaire and a post-concussion symptom scale consisting of 22 commonly reported symptoms. All participants indicated whether they endorsed each symptom and rated the extent of symptom severity on a seven-point Likert scale (0 = ‘not experiencing the symptom’ to 6 = ‘severe’). Scores were summed with higher scores reflecting more post-concussive symptoms. The six neurocognitive ImPACT sub-tests consist of word memory, design memory, X’s and O’s, symbol match, colour match and three letters. Five neurocognitive domain scores are derived from the sub-tests: verbal memory, visual memory, visual–motor processing speed, reaction time and impulse control. Higher scores on the verbal memory, visual memory and visual–motor processing speed composites reflect stronger performance, whereas a higher score on the reaction time composite reflects slower or worse performance. The impulse control composite provides a measure of errors on testing and is used to determine test validity. Test scores were converted to percentile measures and adjusted for age, gender, learning disability and level of education. ImPACT has adequate psychometric properties, including good construct validity^49^ and test–retest reliability.^50^

As part of the RECOS study protocol, study participants completed additional screening and assessment inventories to examine clinical presentation of SRC.^34^ Measures collected include Digit Span,^51^ Digit Symbol Coding and Symbol Search,^52^ a nine-hole peg test of fine motor dexterity^53^ and balance assessments including a virtual reality system,^54^ gait analysis^55,56^ and the modified balance error scoring system (mBESS),^57^ facilitating precise concussion diagnosis. Additional assessments, inventories and neuroimaging components collected by the RECOS protocol are reported in Yakoub *et al*.^34^

### Statistical analysis

To identify between-group differences in regional MSSD_BOLD_ measures and ImPACT module composite scores, statistical unpaired *t*-tests were performed and corrected for multiple comparisons using Benjamini–Hochberg control of the false discovery rate (FDR). These comparisons provide for interpretable identification of the specific ImPACT scores and modules most readily associated with SRC status, assessing for group-level effects of SRC and repetitive mTBI that emerge in variables used by our machine learning framework.

### Random forest machine learning

BOLD variability indices and ImPACT composite scores were further assessed for their ability to differentiate healthy, concussion and repetitive concussion subjects and predict clinical symptom load using a random forest (RF) machine learning approach. RF is an ensemble machine learning method that trains an ensemble (i.e. forest) of decision trees to perform either classification or regression.^58^ RF machine learning achieves high prediction accuracy when presented with a large number of input variables relative to the sample size,^59,60^ as is the case for our neuroimaging data, while still performing well with small numbers of input variables (as is the case for ImPACT scores). This importantly affords the ability to directly compare predictions of concussion status and post-concussion symptoms made by modelling BOLD variability and ImPACT composite scores using the same machine learning approach. Other motivations for selecting RF machine learning for this study include RF’s understood performance, ease of use and resistance to outliers, as well as its capability to consider all BOLD variability regions jointly, avoiding an initial feature selection or engineering step in support of the exploratory nature of this study. Critically, this approach avoids using the results from between-group feature comparisons of BOLD variability to subset regional BOLD variability features that build a smaller and better-performing RF model, as feature engineering BOLD variability metrics is not a goal of the present study.

In our approach, bagged RF regression and classification were performed using built-in functions in MATLAB R2021a. MSSD_BOLD_ measures and ImPACT composite scores were used variously as input features (i.e. independent variables), with output features (i.e. dependent variables) being group membership (classification) or post-concussion symptoms (regression). Each RF ensemble was parameterized using leave-one-out crossfold validation, with the minimum leaf size being the only tuned parameter. For ImPACT score models, minimum leaf size was tuned to 5 (preventing overfitting); otherwise, minimum leaf size was tuned to 20. Trees were grown by splitting input variables to maximize reduction in the Gini impurity index,^61^ repeated iteratively until maximum depth is reached or when all samples belong to a single class. The number of decision trees was increased until mean squared error (MSE) of out-of-bag (OOB) classification or regression predictions stabilized (limiting overfitting and facilitating generalization to test subjects). Forests were grown to 5000 trees total.

### Classification and regression performance of RF models

Individual RF machine learning predictions were generated from an ensemble of OOB decision trees, where the ensemble of decision trees that generate a prediction did not include the test subject’s data in any resampled training data set. Restated, the set of decision trees used to make a prediction for test predicts subject *K* from a model fold (i.e. bag) that has been trained with resampling from all other athletes in our data set *N*, affording a direct test of RF models’ ability to generalize their training data to unseen test set (i.e. OOB) subjects. The overall OOB prediction performance of RF machine learning evaluate three separate sets of input features: (i) ImPACT module composite scores, (ii) whole-brain MSSD_BOLD_ measures and (iii) ImPACT score and MSSD_BOLD_ measures jointly combined. This approach allows us to assess the utility of BOLD variability or ImPACT scores in isolation or combination when predicting SRC status and post-concussion symptoms. One versus all receiver operating characteristic (ROC) curves were calculated for the classification of each group across the three classes of RF models, indicating the true positive rate (i.e. correctly predicting group membership) versus the false positive rate (i.e. incorrectly predicting group membership in the next most likely classification).

The ROC area under the curve (AUC) was computed for all combinations of group and model to diagnose the overall prediction performance of each class. This AUC is the integral of the ROC curve—i.e. the probability that the conditional probability of a random positive observation is greater than the conditional probability of a random negative observation. Thus, larger probabilities represent greater sensitivity and specificity for the positive label, suggesting greater utility for detecting SRC.

For regression models, RF machine learning was instead trained to predict total reported post-concussion symptoms from the same combinations of input BOLD variability and ImPACT score variables (while ignoring group membership). Importantly, control subjects may incidentally report low levels of concussion symptoms in the absence of SRC—further motivating the investigation of functional neuroimaging metrics for detecting SRC status.

## Results

Table 1 reports demographic and clinical characteristics of our sample. All subjects were male semi-professional rugby athletes between the ages of 17 and 31. All concussed athletes had experienced an SRC in the past 2–5 days (average 3.5). Six athletes had experienced repetitive mTBI, experiencing a second prior concussion within a 21-day window.

**Table 1:** Clinical and demographic characteristics of study sample (*N* = sample size; *µ* = mean; *σ* = std. deviation)

Study group	*N*	Age			Days since SRC		
		Μ	*σ*	Range	*μ*	*σ*	Range
Control	15	25.3	2.6	(20–28)			
Concussion	29	23.3	3.4	(17–31)	3.5	1.4	(2–5)
Repetitive concussion	6	23.7	4.0	(18–27)	3.2	1.0	(2–5)

### Between-group effects

#### ImPACT scores

**Figure 1:**
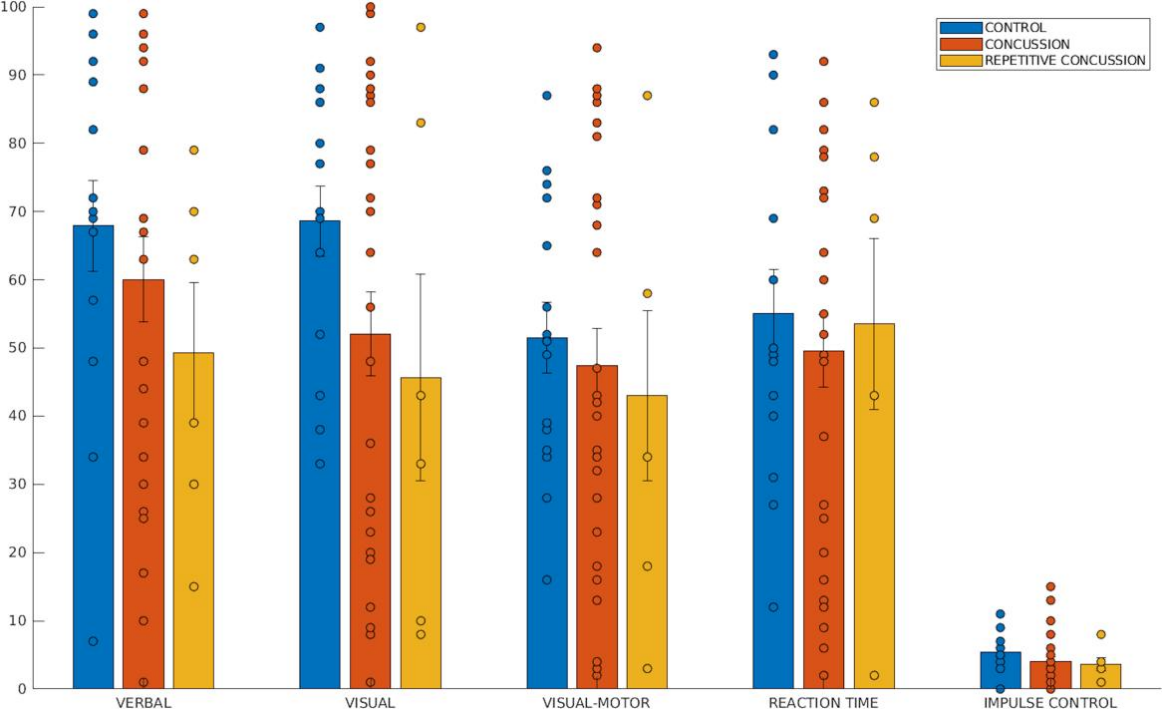
**ImPACT performance.** Descriptive Bar plot of means and standard error bars for ImPACT composite scores in the control group (leftmost bar), concussed group (middle bar) and repetitive concussion group (right bar). Across most test modules, standard error of the mean overlaps between groups. For visual memory scores, concussion group scores are lower than control group scores. Paired *t*-tests show no significant uncorrected (*P <* 0.05) between-group differences for ImPACT composite scores.

**Figure 2:**
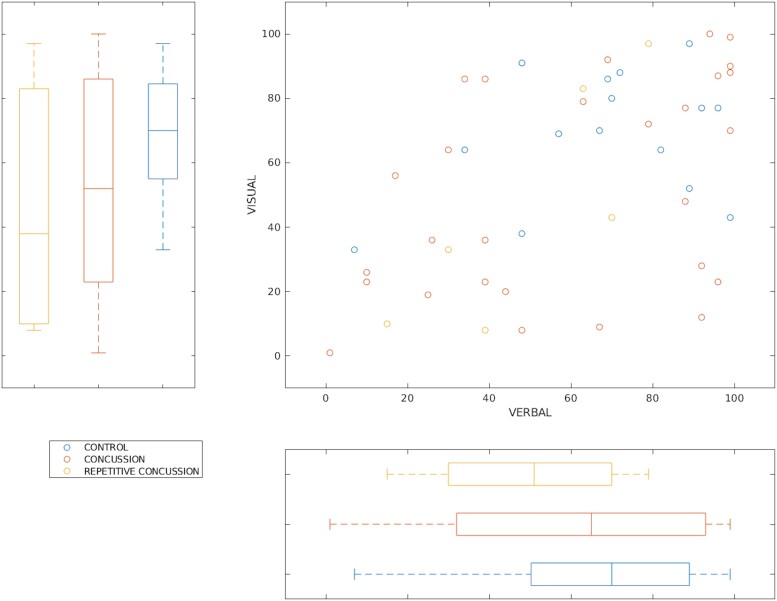
**Between-group module scores.** Group scatter plot with marginal box plots of verbal memory and visual memory score for control group, concussed group, and repetitive concussion group. Paired *t*-tests show no significant (*P <* 0.05) uncorrected between-group differences for ImPACT composite scores.

Statistically significant between-group differences were not observed for ImPACT’s composite measures of verbal memory, visual memory, visual–motor processing speed, reaction time and impulse control between healthy control, concussed individuals and repetitive concussion individuals. Figure 1 shows bar plots of the mean and standard error of the mean (SEM) overlaid with datapoints for each of the module percentile scores across all groups. While there were no reliable pairwise differences in ImPACT module scores between any two conditions, concussed groups had lower mean scores across modules (e.g. Figure 2). None of these mean differences were statistically significant (lowest *P* value = 0.11). Impulse control is a measure of test validity, used to discard tests with a value above 30. The highest observed impulse control score in our sample was 15 (*µ* = 4.4, *σ* = 3.4), suggesting all ImPACT scores represent valid administrations and should be included in our analysis.

#### Regional BOLD variability

Table 2 lists cortical brain areas that display a significant difference in MSSD_BOLD_ measures for concussion or repetitive concussion athletes compared with healthy controls. In contrast with ImPACT score findings, reliable regional differences in MSSD_BOLD_ signal are found in occipital and inferior temporal lobes, premotor areas and the ventral anterior cingulate. We observe that regional MSSD_BOLD_ values are significantly decreased in concussed subjects relative to healthy controls, displaying significantly less fluctuation in BOLD signal amplitude over time. In contrast, all significant regions in repetitive concussion patients show greater MSSD_BOLD_ than in healthy controls, reflecting significantly more pronounced variability in regional BOLD signal relative. This dissociation between BOLD signal decreases in concussion and increases in repetitive concussion suggests that unique pathophysiological mechanisms that implicate functional alterations may underlie the more severe sequelae observed in repetitive SRC.

**Table 2:** Cortical regions with significant differences in MSSD_BOLD_ (two-tailed |*t*| *>* 2.04, *P <* 0.05; FDR) between concussed participants and healthy controls and between repetitive concussion patients and healthy controls (two-tailed |*t*| *>* 2.11, *P <* 0.05; FDR). Columns 1–5 represent region name, *t*-test statistic and the *x*, *y* and *z* centroid coordinates of the respective Schaefer atlas parcellation unit in MNI space. Negative *t*-test statistic implies MSSD_BOLD_ in concussed participants is greater than MSSD_BOLD_ in healthy controls. The opposite is true for positive *t*-test statistic (brain hemispheres are indicated by ‘L’ for left and ‘R’ for right)

	*t*-stat	*x*	*y*	*z*
**Brain region—concussion**				
L. Primary visual cortex (striate)	2.64	−6	−92	−4
L. Preoccipital area	2.04	−24	−88	24
R. Secondary visual cortex	2.66	20	−90	22
R. Premotor area and supplementary motor area	2.21	46	−12	48
R. Fusiform face area	2.12	42	−46	−22
**Brain region—repetitive concussion**				
L. Limbic cortex (VAC)	−2.11	−6	10	42
R. Secondary visual cortex	−2.33	30	−94	−4

#### RF classification

Despite evidence for group-level differences in regional BOLD signal variability, no feature selection was performed prior to training RF classifier models, and therefore, all regional MSSD_BOLD_ metrics and ImPACT scores were used to train RF machine learning models. Table 3 presents the total classification accuracy for RF models trained on ImPACT scores and BOLD variability measures in isolation or combination. RF models trained with a combination of ImPACT scores and MSSD_BOLD_ demonstrated the highest classification accuracy, with ImPACT scores alone producing models with the lowest classification accuracy.

**Table 3:** Random forest classification error (based on OOB decision trees) across all study groups by input data stream

Training data	Out-of-bag Classification error	Classification accuracy
ImPACT scores	46%	54%
BOLD variability	42%	58%
ImPACT scores and BOLD variability	38%	62%

Table 4 lists multi-way classification AUC of RF models for each classification label (i.e. study group). The prior probability of group membership represents the baseline level of classification performance we would expect using group frequencies alone, in the absence of any ImPACT or MSSD_BOLD_ data. Only the model trained on ImPACT scores alone under-performs this baseline value for classifying concussion subjects (consistent with the lack of group differences observed in ImPACT module scores; Fig. 1). In all comparisons, RF models trained using MSSD_BOLD_ measures outperform ImPACT scores alone. We observe only marginal difference between the predictions of RF models trained on MSSD_BOLD_ alone versus MSSD_BOLD_ variability and ImPACT jointly, suggesting that with respect to classification performance, variance in the ImPACT measure is somewhat redundant with BOLD variability. AUC values for the repetitive concussion label are most diagnostic of overall multi-way classification and overall model performance, being the smallest positive label (*N* = 6). Here, we observe the greatest difference in classification performance between ImPACT scores and MSSD_BOLD_ measures, suggesting BOLD variability is both a more sensitive and specific index of signal associated with repetitive SRC than ImPACT scores.

**Table 4:** AUC for random forest classifier’s ability to distinguish group membership by input data stream. The prior probability of a group classification displays expected baseline model performance and AUC in the absence of any subject-level variables

ROC	Prior probability	ImPACT	MSSD_BOLD_	ImPACT and MSSD_BOLD_
Control	0.30	0.59	0.59	0.63
Concussion	0.58	0.52	0.63	0.65
Repetitive concussion	0.12	0.25	0.54	0.66

Table 5 displays the confusion matrix for OOB classifications based on the best-performing RF model (trained using ImPACT scores and MSSD_BOLD_ variability in combination). Ground truth positive labels are reflected along rows, with OOB classification labels in columns (such that values along the main diagonal reflect true positives). Label confusion suggests this best-performing RF model (correctly classifying 31/50 athletes in our sample) is most sensitive for detecting concussion and repetitive concussion. However, the RF model also displays markedly lower specificity for concussion, misclassifying a majority (11/15) of healthy control subjects as concussed. This suggests that while BOLD variability indeed facilitates detection of SRC and repetitive mTBI, models trained to include BOLD variability measures may require more targeted feature engineering to better distinguish between health controls and subjects with lower SRC severity, as has been shown using functional connectivity data.^20^ This model’s performance for classifying control subjects (4/15) is also an improvement over the RF model trained using only ImPACT composite scores, which misclassifies all control subjects as concussion subjects (0/15). This performance likely reflects the large label size of concussion athletes and the lack of reliable between-group differences observed with ImPACT scores. These results suggest that MSSD_BOLD_ measures indeed contribute to a classification model with improved prediction accuracy for distinguishing healthy and concussed individuals.

**Table 5:** Confusion matrix for OOB model classifications trained using ImPACT scores and MSSD_BOLD_ signals in combination. Ground truth positive labels are displayed in rows with classifications in columns

	Control	Concussed	Repetitive
Control	4	11	0
Concussed	0	23	0
Repetitive	6	2	4

#### RF prediction

**Figure 3:**
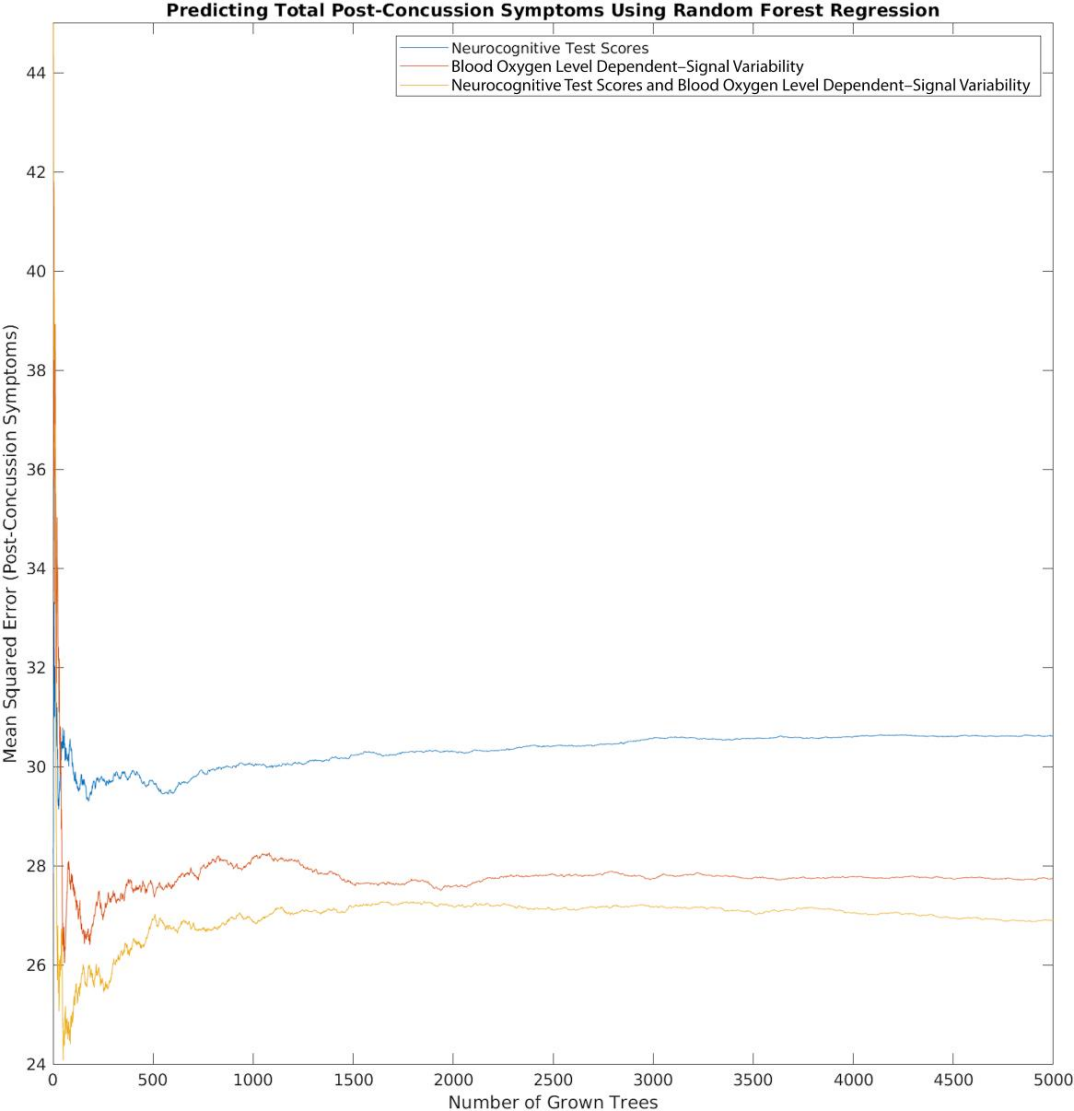
**Predictive model performance.** Random forest model mean squared error (MSE) across model training when predicting self-reported post-concussion symptoms in all athletes.

Table 6 reports MSE of RF regression models trained to predict total post-concussion symptoms reported in the RECOS protocol. Training error was found to be the lowest when models included both BOLD variability measures and ImPACT composite scores as input variables, with the second best training performance observed in models that were trained using only BOLD variability features. In contrast, predictions from the RF model trained on ImPACT scores alone provide the least accurate assessment of self-reported clinical symptomatology. Consistent with the classification results reported in Table 5, these findings highlight BOLD variability’s utility as a potential marker for symptomatic disruptions of brain function in SRC, able to more accurately predict total post-concussion symptoms in both control and concussion subjects compared with ImPACT scores alone. Figure 3 plots levels of MSE observed for each RF regression model.

**Table 6:** RF training prediction error (MSE across OOB samples from 5000 decision tries) for healthy and concussed athletes by input data source

Input data	Converged Out-of-bag MSE
ImPACT scores	30.5
BOLD variability	28.7
ImPACT scores and BOLD variability	26.9

## Discussion

The present study demonstrates that MSSD_BOLD_ holds promise as a sensitive biomarker for detecting symptomatic abnormalities in cerebrovascular status in athletes with SRC. We briefly review the findings with respect to regional MSSD_BOLD_ and ImPACT modules by examining (i) between-group differences, (ii) their classification accuracy using the categorical RF model and (iii) their regression accuracy using the continuous RF model.

### Neuroimaging indices

SRC typically impacts regions within frontal, posterior and lateral cortical regions due to vibration and lateral displacement of brain tissue.^12^ Our results identify several cortical areas in those regions that demonstrate significant differences in MSSD_BOLD_ measures in the presence of SRC or repetitive mTBI. Specifically, MSSD_BOLD_ measures in occipital and inferior temporal regions (e.g. visual cortex and fusiform face area) and midline structures (premotor cortex and anterior cingulate) significantly differ between control and concussed athletes. These regions are particularly vulnerable to injury due to inertial forces, either front to back or rotational. We also observed a dissociation in the directionality of BOLD variability changes. Concussed athletes displayed universally decreased BOLD variability across affected brain regions. In addition to reflecting altered neurovascular coupling, patterns of low regional BOLD variability observed for concussion patients may also in part reflect functional hyperconnectivity commonly observed following mTBI,^62^ such that stronger functional coupling of distal regions entrains BOLD signal amplitude to a more limited band. In contrast, repetitive concussion subjects display uniformly elevated BOLD signal variability in affected regions. As the elapsed duration between most recent SRC and clinical assessment is comparable for concussion and repetitive concussed athletes (Table 1), this dissociation implies unique neurovascular alterations may be induced in repetitive mTBI compared with single mTBI.

The observed regional differences in MSSD_BOLD_ may also reflect disturbances in CBF regulatory mechanisms, such as localized changes in the density and diameter of capillaries and smooth muscle reactivity as a result of brain injury.^30,32,63^ CBF, for example, can have uneven distribution in concussed individuals due to the impact on the skull and subsequent deformation to localized areas.^3^ One study reported an increase in CBF in the frontal and occipital lobes and the striatum post-mTBI,^64^ while others have observed both an increase and decrease in CBF within multiple brain regions following mTBI.^65,66^ These abnormal fluctuations in CBF lend support to the observed deviations in MSSD_BOLD_, although the direction of changes between mTBI and repetitive mTBI remains to be further explored and validated within a larger data set.

### ImPACT

There were no significant pairwise group differences in any of the five ImPACT scores, suggesting that ImPACT composites have low specificity for neurocognitively healthy controls, low sensitivity for diagnosing neurocognitive deficits in SRC and low sensitivity for identifying repetitive mTBI. Models train on ImPACT scores alone performed poorly when classifying concussed subjects, with predictions showing AUC = 0.54 for classifying concussion subjects and AUC = 0.25 for classifying repetitive concussion subjects. Recent findings have demonstrated that ImPACT may not be sufficiently sensitive in measuring neurocognitive deficits in concussed athletes. Several studies indicate that high school, collegiate^67^ and adult^68^ athletes with history of concussion performed similarly on ImPACT with respect to age- and sex-matched controls. Additionally, a study examining cognitive functioning in adult active duty military personnel with and without mTBI found that after controlling for demographic variables, there were no meaningful effects of mTBI on ImPACT sub-tests.^69^ Moreover, several studies revealed that the ImPACT composite scores provided poor predictive accuracy when distinguishing concussed rugby union athletes,^68^ adolescents^70^ and adults^71^ from controls.

### RF model classification predictions

Including BOLD variability data and ImPACT in categorical RF models produced the highest classification accuracy across all groups (mean AUC = 0.65). In these models, the majority of features ranked as highly important represented BOLD variability measures, not ImPACT scores. Classification based on the categorical RF models demonstrated a higher sensitivity or true positive rate for accurately identifying individuals with repetitive concussions when using BOLD variability (AUC = 0.66) than was observed using ImPACT scores (AUC = 0.25). Overall multi-label classification accuracy was also increased when BOLD variability data were included, with classification error decreasing from 46% (based on ImPACT scores alone) to 38% when ImPACT scores and BOLD variability were jointly modelled. This was partially due to increases in classification accuracy for identifying subjects with high overall symptom loads as belonging to concussion or repetitive concussion groups. Classification accuracy of control subjects, while still low, also increased when BOLD variability data were included in the RF models, suggesting that BOLD variability may have greater specificity than ImPACT for detecting SRC. The left middle frontal gyrus, left frontal pole and temporal occipital fusiform gyrus had the largest effects in the RF model classification, which is in line with research suggesting regions in the frontal and temporal lobes are commonly affected in mTBI.^72^ Similar inferior temporal and occipital regions were observed in pairwise FDR-corrected neuroimaging indices between groups, and RF modelling identified additional predictive regions in frontal areas. Given their proximity to bony protuberances inside the skull, these regions are vulnerable to coup–contrecoup injury, which may account for observed MSSD_BOLD_ abnormalities in this analysis. Results from the RF model indicate that MSSD_BOLD_ is an important parameter for characterizing disruptions in cerebral perfusion in SRC and could potentially provide a biomarker for injury detection.

### RF model regression predictions

Prediction accuracy was highest for RF models trained to predict the total number of post-concussion symptoms using the combination of ImPACT composite scores and BOLD variability variables. The continuous prediction model trained only on ImPACT scores converges during training to a MSE of 30.5 when predicting post-concussion symptoms, showing the largest error and the worst performance. A continuous RF model that trained only on BOLD variability data demonstrated lower MSE (28.7), reflecting improvements to predictive performance. Jointly including ImPACT and MSSD_BOLD_ data in a single model produced the best prediction performance with a MSE of 26.9. Feature importance scores indicated that the main variables used to generate predictions for this model were BOLD variability measures, suggesting that variance associated with ImPACT scores is of lower importance than BOLD variability during forest growth and model training. Results from these continuous RF models indicate that MSSD_BOLD_ is a more sensitive predictor of post-concussion symptom totals and that jointly considering functional and neurocognitive data together is a useful approach for machine learning models to improve the accuracy of predictions for total SRC symptom load.

### Limitations

In this study, we demonstrate that BOLD signal variability holds promise as a diagnostic biomarker for detecting functional neural alterations following SRC. While our approach motivates systematic exploration into BOLD variability’s utility for detecting injury in clinical neuroimaging of concussion, there are several limitations and challenges to this approach. First, given that the concussed group is a highly homogenous athletic population, future studies should examine whether the observed deviations in MSSD_BOLD_ can be generalized to other populations (i.e. children and adolescents with SRC and non-athletic populations with mTBI). Second, MSSD_BOLD_ cannot currently be used to assess severity of an injury itself, only the extent of the symptom load that it produces, and additional screening methods involving structural neuroimaging techniques will be still required to reveal the extent of injury and changes in pathology during recovery. Third, the analysis approach using MSSD_BOLD_ cannot reveal the actual damage to the vasculature and surrounding neural cells at the site of injury. Fourth, while our study observes high performance for predicting total clinical symptom load in healthy and SRC athletes, our SRC classification models will often mistake healthy controls for concussed subjects. This may be due to our sample’s imbalance between control (*N* = 15) and concussion (*N* = 29) subjects or may suggest that more a more precise feature selection step is required to identify MSSD_BOLD_ regions more specific for SRC, possibly requiring higher-resolution cortical parcellation schemes. On the other hand, mismatches in performance may potentially be addressed by methods that do heavily subset BOLD variability as an initial feature selection step, potentially inducing parity in the number of BOLD and ImPACT input variables to afford comparison of prediction accuracy through alternative ML approaches (e.g. single decision trees, logistic regression, naive Bayes or Bayesian causal models). While random forests appear to be sufficient for our purposes in this manuscript, we do not intend to suggest they are necessary. Fifth, there may be other representations of variability besides MSSD_BOLD_ that better capture the extent of underlying functional alterations in SRC, including representations that jointly consider resting static or dynamic functional correlation or large-scale intrinsic brain network organization. Future research should consider investigating these elements at a fine-grained level to further elucidate neurobiological pathologies in SRC. Sixth, and finally, functional MRI acquisition remains prohibitive in many outpatient clinical settings, though we believe our results at do least motivate further exploration of BOLD variability for monitoring athletes before and after SRC, particularly from a personalized or precision medicine perspective.

These findings emphasize the need for future research to examine how network topology and dynamics are altered by TBI. Recent research has highlighted the importance of global brain network topology for facilitating cognitive abilities,^73^ consistent with the present findings. Executive dysfunction can be a pernicious consequence of TBI,^74,75^ implicating alterations to network topology and dynamics in neurological sequela following mTBI.^76^ Future research may wish to examine brain network alterations for their impact on other cognitive domains or as a potential neurobiological marker of injury and recovery status.

## Conclusion

Diagnosis of SRC can be difficult due to the low sensitivity of assessments designed to identify cognitive deficits in athletes with suspected injuries. In addition, clinical evaluation of SRC using structural imaging methods is limited to characterizing anatomic pathology and cannot detect haemodynamic or neurometabolic disruptions. In this work, we provide evidence that functional neuroimaging indices of MSSD_BOLD_ assessed from resting-state fMRI are sensitive to abnormalities in cerebrovascular status in athletes with SRC. Further, we show that regional MSSD_BOLD_ measures can more sensitively and specifically identify concussed and repetitive concussed individuals within a RF machine learning framework compared with standardized neurocognitive tests. In contrast, ImPACT composite scores do not capture reliable between-group differences in concussion status and cannot be used in isolation to build an RF model that discriminates between concussion, repetitive concussion and healthy athletes. Combining BOLD variability and ImPACT scores leads to the highest prediction performance, supporting the claim that altered BOLD variability provides novel information about altered cerebral vascular status that can aid diagnosis and return-to-play decisions when collected alongside existing assessment protocols for the detection of SRC. Further, we provide evidence that MSSD_BOLD_ contributes to prediction of total post-concussion symptoms, outperforming ImPACT scores across healthy and concussed patients. In conclusion, this paper provides initial evidence that BOLD variability holds promise as a potential clinical marker for SRC, motivating a more systematic exploration of BOLD variability as a candidate biomarker in future work. To investigate the sensitivity and efficacy of MSSD_BOLD_ in diagnosing SRC, future neuroimaging research may wish to deploy MSSD_BOLD_ measures alongside neuropsychological testing protocols and structural neuroimaging methods, providing a more complete assessment of athlete health that includes cerebrovascular alterations and associated cognitive deficits to better facilitate detection of injury in SRC and guide return-to-play decisions.

## Data Availability

Deidentified data supporting the findings of this study may be made available by the authors on reasonable request.
